# Retrograde recanalization of native right coronary artery chronic total occlusion (CTO) through left coronary artery CTO after bypass graft failure: A case report

**DOI:** 10.1097/MD.0000000000020850

**Published:** 2020-07-10

**Authors:** Qing Qin, Jianying Ma, Junbo Ge

**Affiliations:** Department of Cardiology, Zhongshan Hospital, Fudan University, Shanghai Institute of Cardiovascular Disease, Shanghai, China.

**Keywords:** coronary artery bypass grafting, retrograde percutaneous coronary intervention, chronic total occlusion

## Abstract

**Rationale:**

With the development and standardization of modern chronic total occlusions (CTOs) recanalization technique, percutaneous coronary intervention has become a promising treatment alternative to surgery after bypass graft failure. Treatment of a native coronary CTO lesion is preferable to treatment of a saphenous vein graft (SVG) CTO supplying the same territory; however, technical expertise is required.

**Patient concerns:**

This is a 69-year-old male with prior history of coronary artery bypass grafting presented with severe dyspnea at mild exertion (NYHA III) of 2 months duration.

**Diagnosis:**

The patient was diagnosed as heart failure caused by ischemia after SVG failure (SVG to right coronary artery) according to electrocardiogram, plasma N-terminal pro-B-type natriuretic peptide levels, and coronary angiogram.

**Interventions:**

We recanalized native right coronary artery CTO by retrograde approach using septal collaterals by surfing technique after recanalization of totally occluded left coronary artery.

**Outcomes:**

Dyspnea was relieved at discharge. At 6-month follow-up, the patient had no recurrence of dyspnea.

**Lessons:**

In case of SVG failure, percutaneous coronary intervention of native vessel should be considered as a treatment option. Retrograde approach through native vessel is safe but has requirements for operators’ volume, skill, and experience.

## 1 Introduction

The prevalence of chronic total occlusions (CTOs) in patients with previous coronary artery bypass grafting (CABG) is particularly high. With the development and standardization of modern CTO recanalization technique, percutaneous coronary intervention (PCI) has become a promising treatment alternative to surgery after bypass graft failure. In these patients, treatment of a native coronary CTO lesion is preferable to treatment of a saphenous vein graft (SVG) CTO supplying the same territory, as antegrade flow may not be frequently restored due to distal embolization and even if SVG PCI is successful, the long-term SVG patency is low.^[1]^ During PCI of native coronary CTO lesion, although SVG or internal mammary artery (IMA) graft can be used for the retrograde approach, the procedure risk is high, as even a small dissection in the graft may cause critical complications.^[1,2]^ Therefore, septal or epicardial collaterals are preferred during retrograde approach. However, patients underwent CABG usually had diffuse lesion or even multivessel CTO lesions in native coronary artery, which makes retrograde approach more complicated. We present a case of retrograde intervention of right coronary artery (RCA) CTO lesion via septal collateral by surfing technique after recanalization of totally occluded left coronary artery.

## 2 Case report

A 69-year-old male with prior history of CABG presented with severe dyspnea at mild exertion (NYHA III) of 2 months duration was admitted in our center. The electrocardiogram showed ST depression in leads II, III, aVF, and V4-6, and blood examination revealed elevation of plasma N-terminal pro-B-type natriuretic peptide levels (2640 pg/mL). Echocardiogram showed left ventricular systolic dysfunction and low left ventricular ejection fraction (30%). The patient had inferior ST-segment-elevation myocardial infarction in 2009, when he was 59 years old, with angiographic evidence of severe 3 vessels disease (coronary angiography showed CTO in proximal left anterior descending artery (LAD), 90% stenosis in mid and distal left circumflex artery, and 95% stenosis in mid RCA. The patient underwent CABG with left internal mammary artery (LIMA) to LAD, and sequential SVG to 1st obtuse marginal branch (OM1), 2nd obtuse marginal branch (OM2), and posterolateral branch (PL) in 2009.

**Figure 1:**
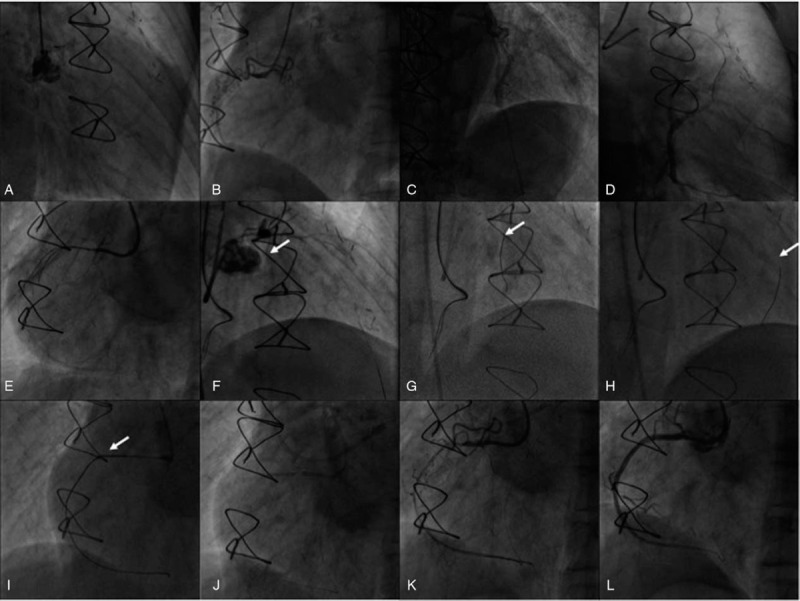
Retrograde recanalization of native right coronary artery chronic total occlusion (CTO) through left coronary artery CTO after bypass graft failure. (A) Totally occluded left coronary artery. (B) Right coronary artery (RCA) CTO with bridging collaterals. (C) Patent of left internal mammary artery to left anterior descending artery (LAD) graft. (D) Patent saphenous vein graft (SVG) to obtuse marginal branch 1 (OM1), OM2 conduits and a complete occlusion of sequential SVG to posterolateral branch conduit. (E) Failed antegrade approach using parallel wire technique with 2 Gaia 3 guidewires. (F) CAG after recanalization of left main and LAD showed proximal septal branch (white arrow). (G) Septal surfing technique trying proximal septal branch (white arrow). (H) Sion guidewire finally entered distal RCA by septal surfing technique using distal septal branch (white arrow). (I) Gaia 3 wire crossed CTO lesion retrogradely into the true lumen in proximal RCA, and was advanced into Guidezilla guide extension catheter (white arrow) positioned in the antegrade guiding catheter. (J–L) Predilation by a 2.0 × 15 mm balloon and stented with 2 overlapping drug-eluting stents (2.5 × 38 mm and 3.0 × 38 mm) with excellent angiographic result and TIMI3 flow in all distal branches.

Coronary angiography was performed via 6 French (Fr) left radial artery access and demonstrated patency of LIMA to LAD and SVG to OM1, OM2 conduits, but a complete occlusion of sequential SVG to PL conduit. Native left main coronary artery was occluded in ostium and native RCA was occluded in the mid portion with bridging collaterals (Fig. 1A–D). We decided to treat the native RCA CTO. Dual arterial access was achieved with another 6 Fr sheath in right femoral artery. The left and right coronary arteries were intubated with 6 Fr AL 0.75 (Launcher; Medtronic; USA) and 6 Fr EBU 3.5 (Launcher; Medtronic; USA) guide catheters, respectively. An antegrade approach via left radial artery was attempted; however, neither Fielder XTR wire (Asahi Intec, Japan) nor Gaia 3 wire (Asahi Intec, Japan) with Finecross microcatheter (Terumo, Japan) reached the true lumen in distal RCA. Then, parallel wire technique with Crusade microcatheter (Kaneka, Japan) and two Gaia 3 wires (Asahi Intec, Japan) were attempted, but also failed (Fig. 1E). We therefore switched to the retrograde approach using septal channel from LAD through occluded left coronary artery. Gaia 3 wire (Asahi Intec, Japan) crossed occluded left main (LM) and LAD, and finally reached true lumen in distal LAD. Sion wire was exchanged by Finecross microcatheter (Terumo, Japan) into dital LAD, and dilation of LM and proximal LAD with a 2.0 × 15 mm balloon was performed (Fig. 1F). Then, septal surfing technique (SST) was used for septal crossing. We tried different septal channels originating from proximal to distal LAD (Fig. 1G, H), and delivered Sion wire (Asahi Intec, Japan) retrogradely through distal septal branch into distal RCA supported by a 150-cm Finecross microcatheter (Terumo, Japan). Gaia 3 wire (Asahi Intec, Japan) crossed CTO lesion retrogradely into the true lumen in proximal RCA, and was advanced into Guidezilla guide extension catheter (Boston Scientific, USA) positioned in the antegrade guiding catheter (Fig. 1I). The Finecross microcatheter (Terumo, Japan) was delivered to the antegrade catheter and a RG3 wire (Asahi Intec, Japan) was externalized. The CTO was then predilated by a 2.0 × 15 mm balloon and stented with 2 overlapping drug-eluting stents (2.5 × 38 mm and 3.0 × 38 mm) with excellent angiographic result and TIMI3 flow in all distal branches (Fig. 1J–L).

Dyspnea was relieved at discharge. At 6-month follow-up, the patient had no recurrence of dyspnea.

## 3 Discussion

In this case, the culprit lesion was the SVG-RCA graft. Three options could be selected: First, redo CABG; 2nd, PCI of the occluded SVG; 3rd, PCI of the native RCA CTO lesion. Recanalization of native CTO vessel in post-CABG patients is often the favored revascularization strategy as redo surgery bears high peri-operative adverse events, and PCI in diseased graft is associated with high thrombus and distal embolization risk.^[3]^ Therefore, we decided to treat native RCA CTO. However, CTO PCI in post-CABG patients is challenging^[4]^ as post-CABG status is associated with accelerated atherosclerosis progression including multivessel CTOs,^[5]^ higher degree of calcification, and negative remodeling.^[6]^ Therefore, the procedure success was lower in CTO PCI in post-CABG patients compared with non-CABG patients.^[3,4]^ As the majority of distal segments of CTO exhibited a tapered pattern, it is easier to place wire in the true lumen by retrograde approach compared with antegrade approach.^[6]^ Also, several studies have demonstrated higher use of retrograde approach in CTO PCI in post-CABG patients compared with CTO patients without prior CABG.^[7,8]^ As a result, we changed to retrograde approach after failure in antegrade approach.

There are 3 options for retrograde approach, including SVG, LIMA-septal collaterals, and LAD-septal collaterals. CTO PCI through retrograde approach via SVG achieved high success rate in previous study.^[9]^ However, in this case, the patient would be at high risk if SVG were injured during PCI as SVG-OM1-OM2 also supplies lateral wall. Similarly, if LIMA was used during PCI and injured, the flow disturbance to the LAD may occur. Therefore, LAD-septal collaterals were chosen to ensure the safety of the procedure regardless of high technical requirement for the operator during PCI as LAD CTO has progressed to LM CTO ten years after CABG and recanalization of LM and LAD should be performed before retrograde approach of RCA PCI. Luckily, Gaia 3 guidewire crossed occluded LM into true lumen of LAD and antegrade flow in LAD was obtained after dilation by a small balloon. Only 1 septal branch was visible by CAG and SST was used to find the collateral connecting distal RCA. SST was used in retrograde approach in search of a path of low resistance by probing septal collaterals by “trial and error” without distal tip injections. SST is safe and feasible and is associated with high collateral crossing regardless of the angiographic appearance of collateral connections.^[10]^ In this case, several invisible septal branches were attempted and finally one of them was crossed by Sion guidewire.

According to previous experience, when native coronary artery was opened through patent SVG by retrograde approach, the newly opened vessel and SVG may be occluded due to competitive flow. Usually, patent SVG should be coiled to prevent occlusion of recanalized native coronary artery.^[9]^ In our case, proximal LAD was dilated with 2.0 mm balloon and only TIMI 1 flow was achieved on CAG, which will not lead to competitive flow and LIMA graft occlusion. Therefore, we did no more intervention to LAD. There is great possibility that proximal LAD will be occluded soon; however, we may recanalize it again if LIMA graft failure occurred in the future.

## 4 Conclusions

In case of SVG failure, PCI of native vessel should be considered as a treatment option. Retrograde approach through native vessel is safe but has requirement for operators’ volume, skill, and experience.

## Author contributions

Original draft preparation and review & editing, QQ; Conceptualization, MJY; Supervision, MJY and GJB

**Conceptualization:** Jianying Ma.

**Supervision:** Jianying Ma, Junbo Ge.

**Writing – original draft:** Qing Qin.
